# Supplementary material for: Toward atomistic models of intact severe acute respiratory syndrome coronavirus 2 via Martini coarse‐grained molecular dynamics simulations
Source: Quant Biol. 2023 Nov 28;11(4):421–33. doi: 10.1002/qub2.20 (PMC12807059; doi:10.1002/qub2.20)
Supplement: Supplementary file 1 — Supporting Information S1 [file QUB2-11-421-s001.pdf]

Supplementary Information for:

Toward atomistic models of intact severe acute respiratory syndrome coronavirus 2 via Martini coarse-grained molecular dynamics simulations

Dali Wang[1,2,†], Jiaxuan Li[1,†], Lei Wang[1], Yipeng Cao[3,4], Bo Kang[4], Xiangfei Meng[4], Sai Li[2,5,*], and Chen Song[1,2,*]

[1]Center for Quantitative Biology, Academy for Advanced Interdisciplinary Studies, Peking University, Beijing 100871, China

[2]Peking-Tsinghua Center for Life Sciences, Beijing 100871, China

[3]Tianjin Medical University Cancer Institute and Hospital, National Clinical Research Center for Cancer, Tianjin 300060, China

[4]National Supercomputer Center in Tianjin, Tianjin 300457, China

[5]Beijing Frontier Research Center for Biological Structure, State Key Laboratory of Membrane Biology, School of Life Sciences, Tsinghua University, Beijing 100084, China

[†]These authors contributed equally to this work.

[*]E-mail: c.song@pku.edu.cn (Chen Song), sai@tsinghua.edu.cn (Sai Li)

1 Supplementary Tables

Table S1. The glycan types of the glycosylated sites of the S protein

	Chain A	Chain B	Chain C
N17, N149<br>N1098	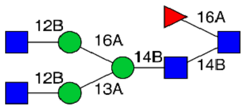	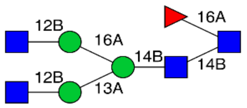	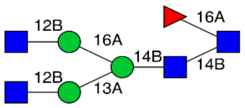
N61, N603<br>N709, N717	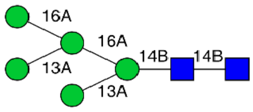	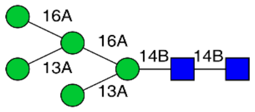	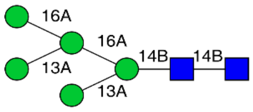
N74	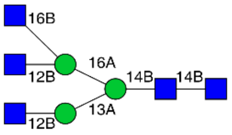	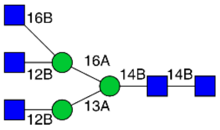	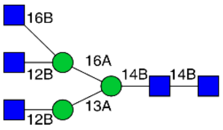
N122, N801	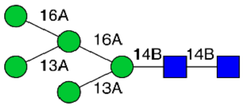	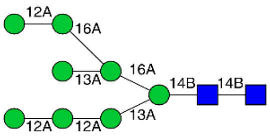	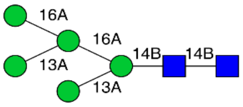
N165	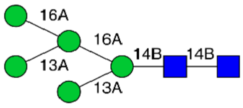	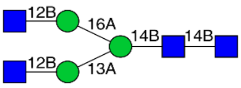	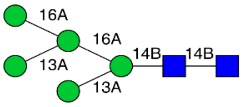
N234	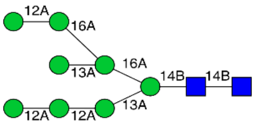	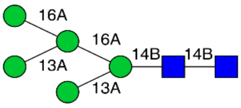	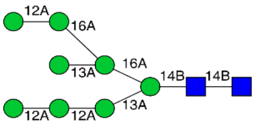

GlcNAc Mannose Fucose

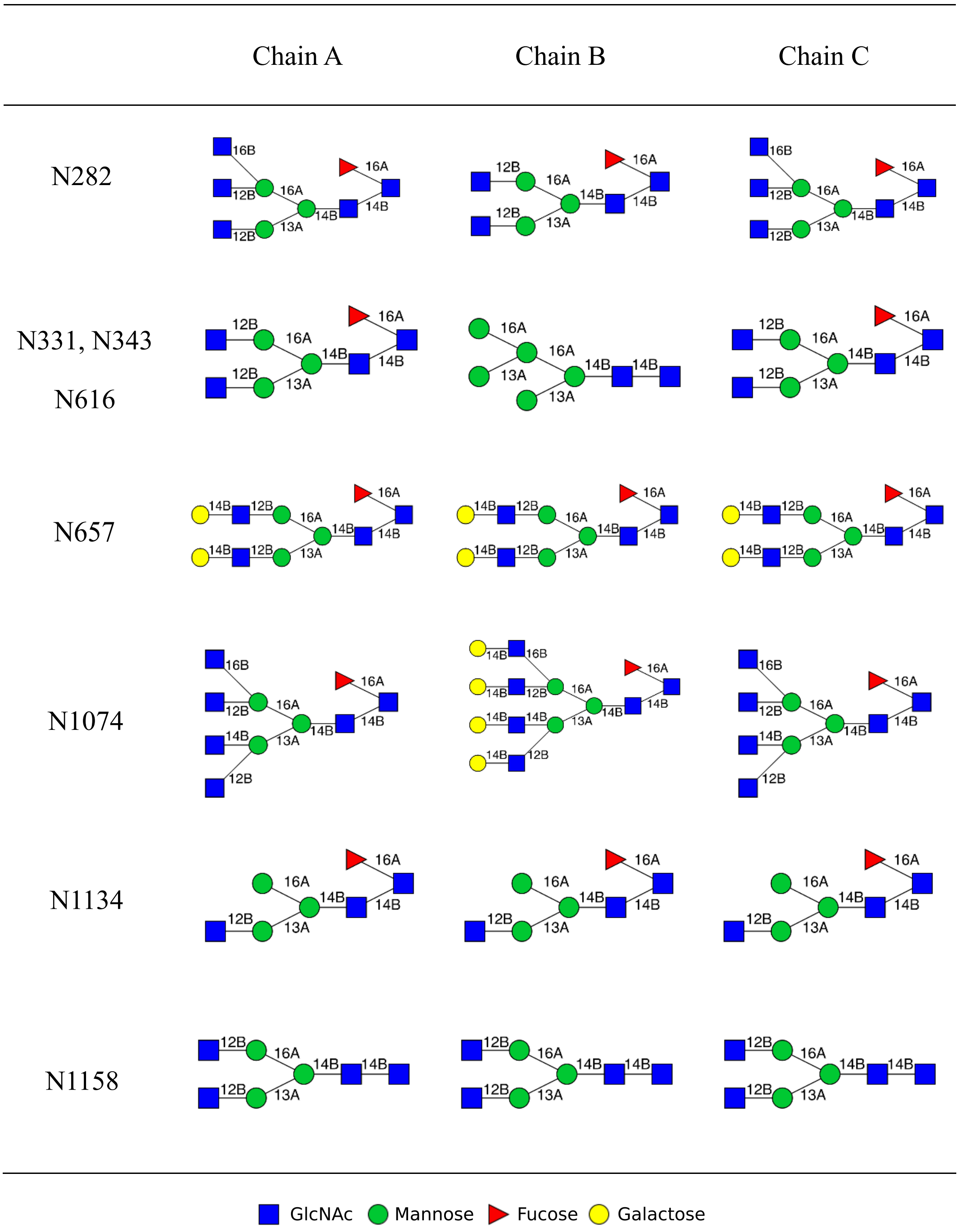
Chain A
Chain B
Chain C
N282
N331, N343
N616
N657
N1074
N1134
N1158
GlcNAc
Mannose
Fucose
Galactose

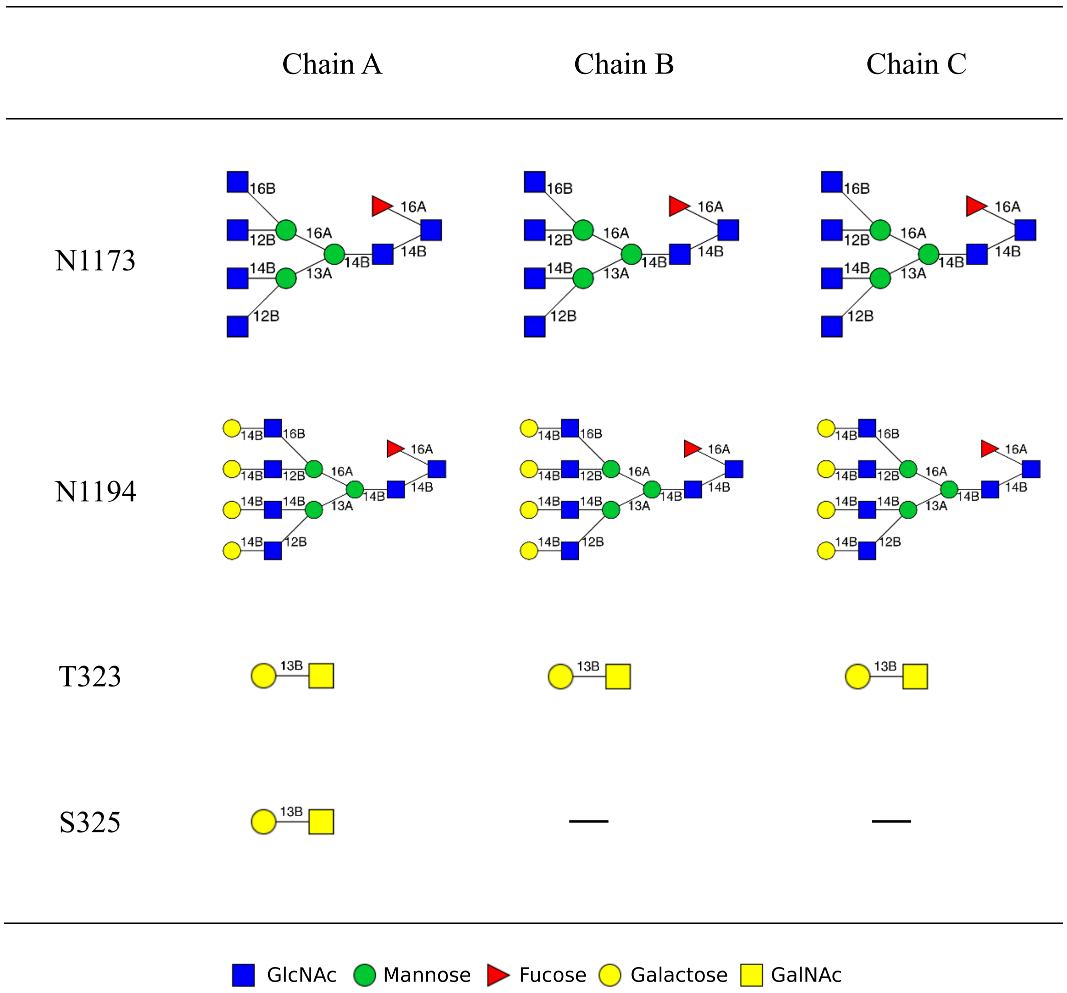
Chain A
Chain B
Chain C
N1173
N1194
T323
S325
GlcNAc
Mannose
Fucose
Galactose
GalNAc

Table S2. The virion system composition

Viral Envelope				
Component	Amount	Numbers in CG beads	Numbers in AA atoms	
1,2-dipalmitoyl-sn-glycero-3-phosphocholine (DPPC)	16,281	195,372	2,116,530	
1-palmitoyl-2-oleoyl-sn-glycero-3-phosphocholine (POPC)	16,284	195,408	2,182,056	
1,2-dipalmitoyl-sn-glycero-3-phosphoethanolamine (DPPE)	7,398	88,776	895,158	
1-palmitoyl-2-oleoyl-sn-glycero-3-phosphoethanol (POPE)	7,409	88,908	926,125	
1,2-dipalmitoyl-sn-glycero-3-phosphoinositol (DPPI)	1,463	20,482	194,579	
1-palmitoyl-2-oleoyl-sn-glycero-3-phosphoinositol (POPI)	1,454	20,356	199,198	
1,2-dipalmitoyl-sn-glycero-3-phosphatidylserine (DPPS)	3,691	44,292	453,993	
1-palmitoyl-2-oleoyl-sn-glycero-3-phosphatidylserine (POPS)	3,711	44,532	471,297	
Cholesterol (CHOL)	11,255	90,040	832,870	
Sphingomyelin (SM)	3,715	40,865	471,805	
Structural proteins				
Name	Amount	Numbers in CG beads	Numbers in AA atoms	Polymerization
Spike_up (S_up)	25	211,425	1,474,925 (none-glycosylated)<br>1,801,550 (full-glycosylated)	Trimer
Spike_down (S_down)	25	211,500	1,475,475 (none-glycosylated)<br>1,802,100 (full-glycosylated)	Trimer
Membrane (M) protein	252	253,512	1,818,432	Dimer
Nucleocapsid (N) protein	160	177,280	1,247,680	Dimer
Envelope (E) protein	2	1,680	12,150	Pentamer
Viral RNA				
Sequence	Amount	Numbers in CG beads	Numbers in AA atoms	
UCUCUAAACG	320	20,160	100,800	
Total		1,704,588	14,873,073 (none-glycosylated)<br>15,526,323 (full-glycosylated)	

2 Supplementary Figures

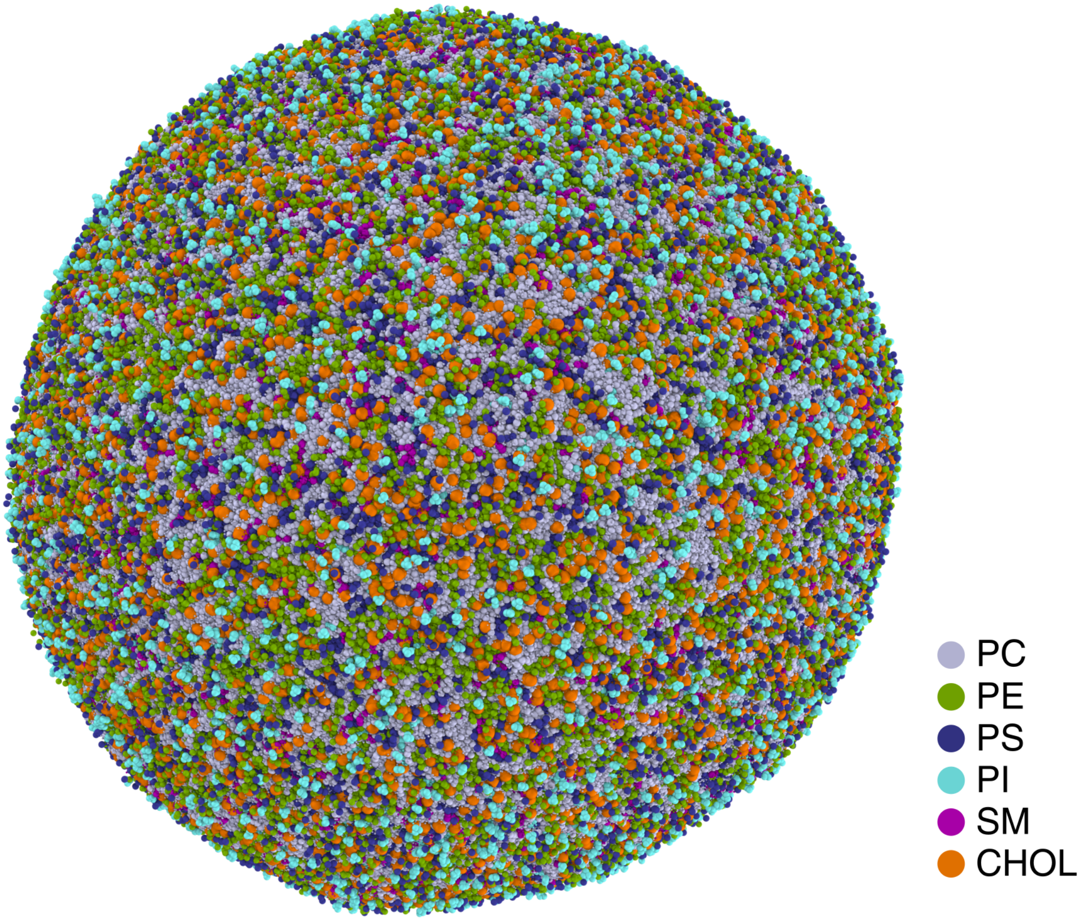

Figure S1: The overview of the equilibrated vesicle.

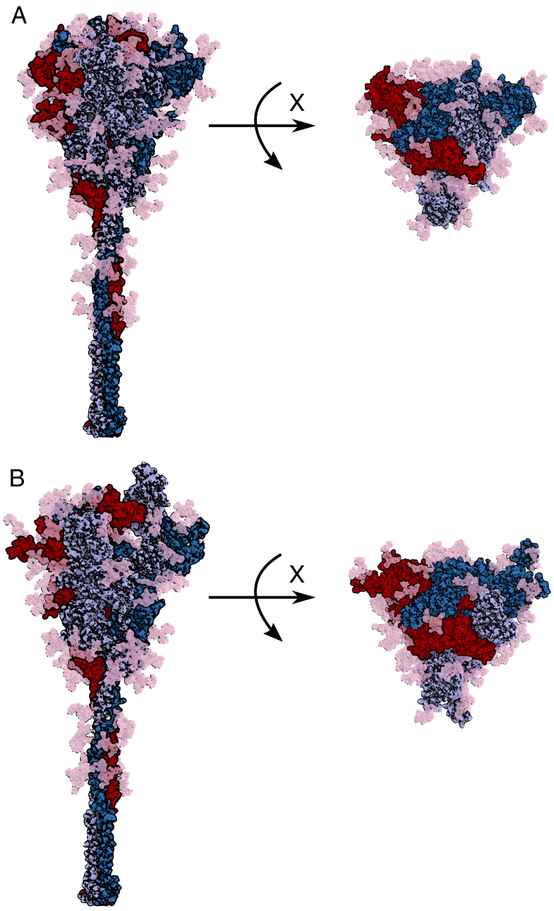

Figure S2: Structures of the spike protein. (A) Full-length "RBD down" state. The three chains were colored blue-white, sky blue, and red, respectively. The glycans were colored light pink. (B) same as (A) but for the "one RBD up" state.

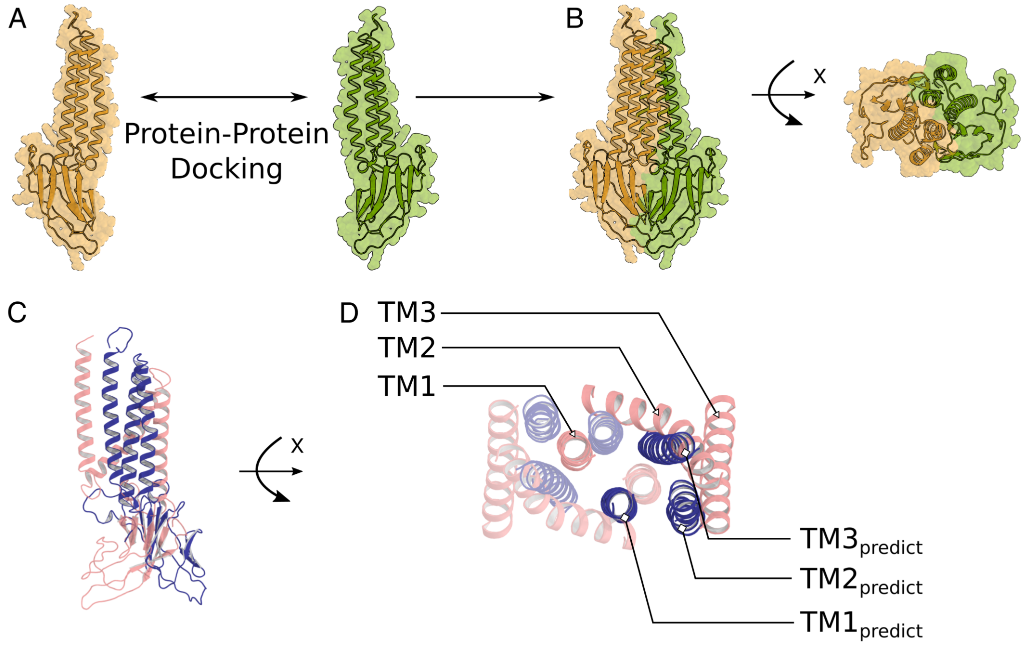

Figure S3: (A) The M protein monomer structure (green and orange) predicted by AlphaFold2; (B) The side and top views of the M dimer structure generated by Protein–Protein docking (ZDOCK). (C) The side view of the two M protein monomer structures: structure from docking (deep blue); "long" structure (pink, PDB ID: 7VGR). (D) The top view of these two M dimer structures transmembrane helices.

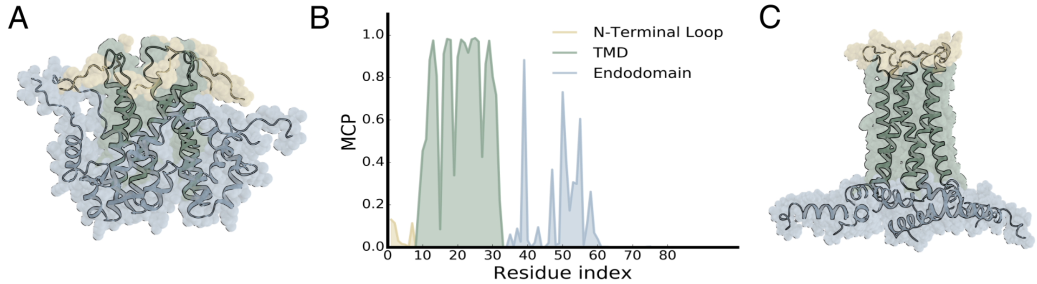

Figure S4: The structures of the E protein pentamer. (A) The homology modeling structure based on the SARS-CoV E protein; (B) The predicted membrane contact probability (MCP) of the E protein. (C) The E protein pentameric structure optimized by Feig's Lab. The brown, green, and blue regions represent the N-terminal loop, the transmembrane domain, and the endodomain of the E protein, respectively.

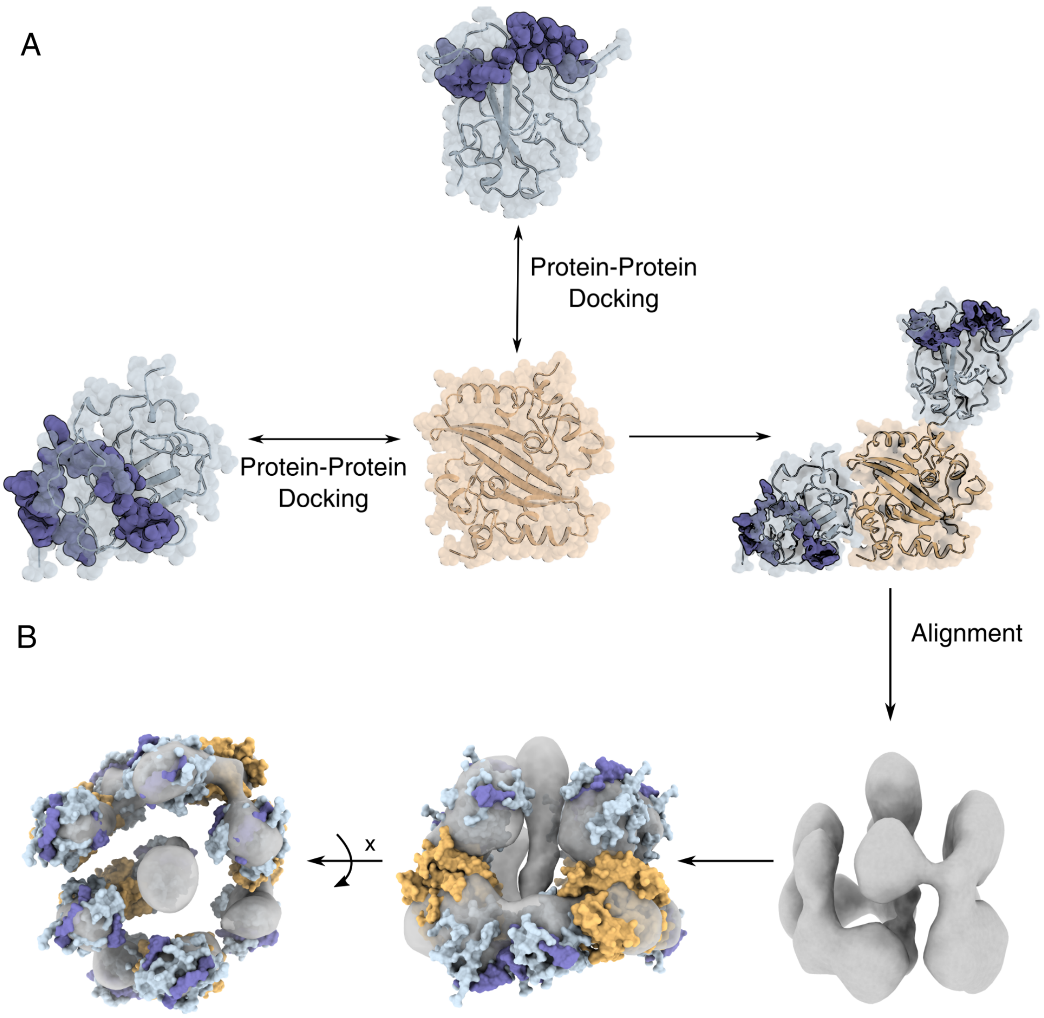

Figure S5: Construction of the RNP unit structure. (A) The N protein dimer (right panel) was generated by docking two NTD structures (light blue) to the dimerized CTD structure (light orange). The RNA segments bound to N proteins were colored deep and opaque blue; (B) Five N protein dimers are fitted into the Cryo-ET density (grey surface) to form a single RNP unit (left panel).

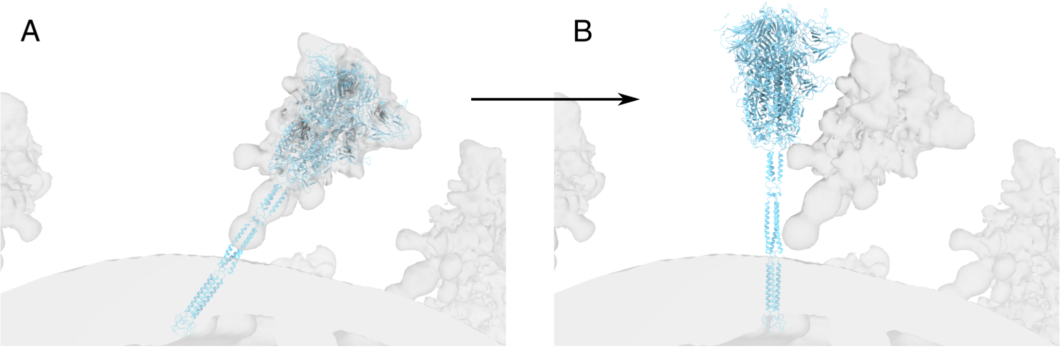

Figure S6: (A) Aligning the S protein into the Cryo-ET density (Chimera –> Fit into density) may lead to inappropriate positioning of the transmembrane domain of the S protein. (B) The adjusted S protein orientation with the transmembrane domain properly embedded into the lipid bilayer. The light blue cartoon indicates the structure of the S protein. The grey surface shows the Cryo-ET density.

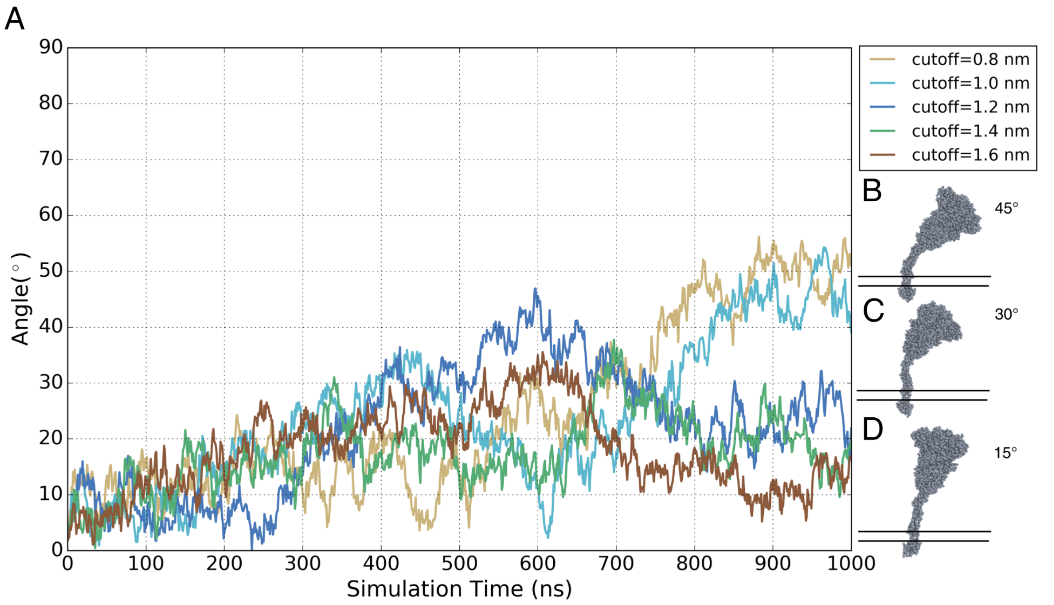

Figure S7: The orientation of the spike protein on the lipid bilayer depends on the elastic network cutoff. (A) Each curve elucidates the spike protein orientation distribution in CG MD simulations with different elastic network cutoff values. The angle was measured between the axis of the spike protein and the normal axis of the membrane surface. (B)–(D) The typical S protein orientations during the simulations.

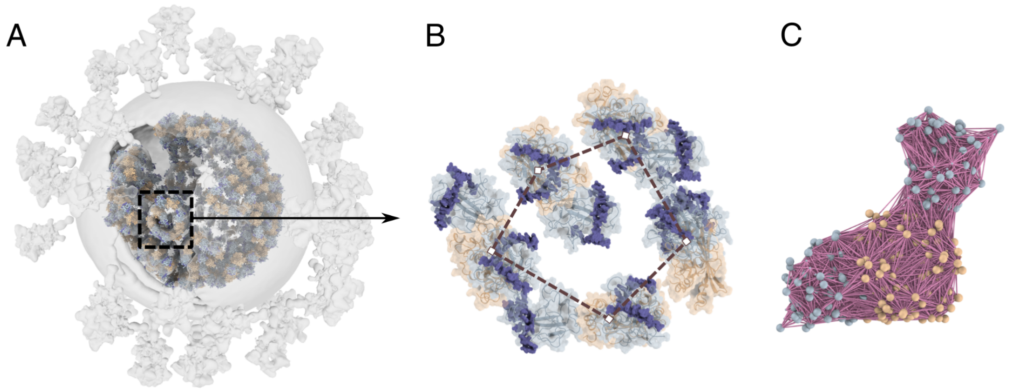

Figure S8: The RNPs and the distance restraints within them. (A) Alignment of the RNP units into the Cryo-ET density map. (B) The zoom-in view of the black frame in (A). The dashed lines represent the distance restraints applied to the N protein dimers in each RNP unit. (C) Magenta lines indicate the pairwise elastic network to maintain the N protein dimer conformation. Light blue and light orange represent domains near the NTD and CTD of the N proteins, respectively. Deep blue indicates the RNA segments bound to N proteins.

3 Supplementary Video

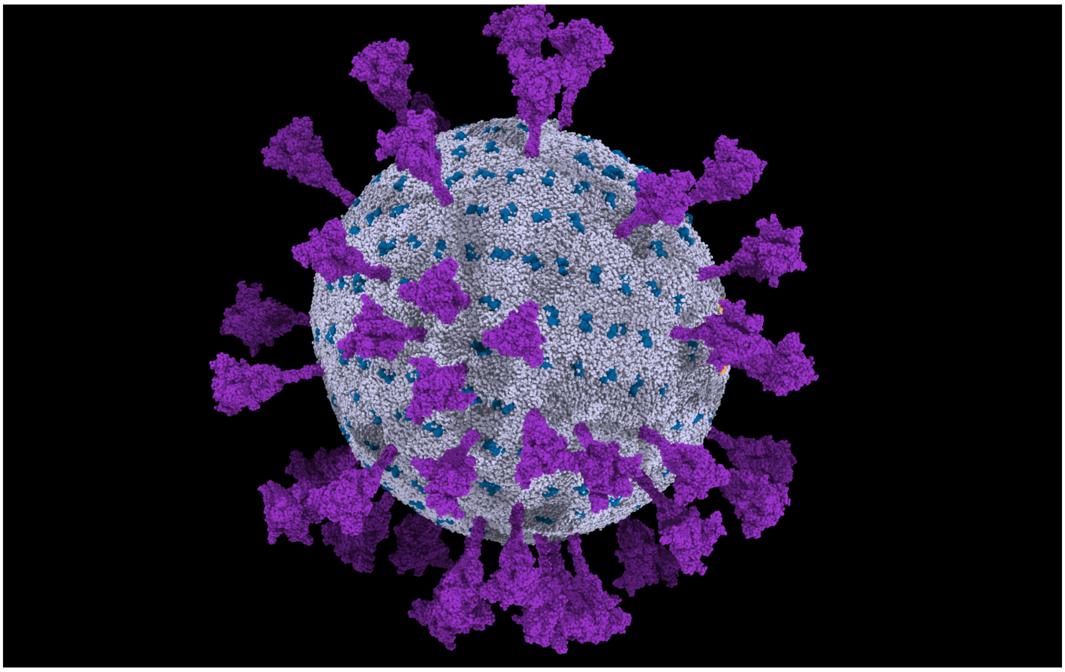

Video S1: Overview of the structural model of SARS-CoV-2 and a dynamic trajectory rendered from Coarse-grained (CG) MD simulations. The CG MD simulation was 500 ns in total, while only the first 200 ns was shown in the video.
